# Unveiling Insights into Ovarian Cancer Metabolism through Space- and Time-Resolved Analysis

**DOI:** 10.3390/cancers15194881

**Published:** 2023-10-08

**Authors:** Wei Jia, Mengci Li

**Affiliations:** 1 School of Chinese Medicine, Hong Kong Baptist University, Kowloon Tong 999077, Hong Kong; 2 Center for Translational Medicine and Shanghai Key Laboratory of Diabetes Mellitus, The Sixth People’s Hospital Affiliated to Shanghai Jiao Tong University School of Medicine, Shanghai 200233, China; limengci@sjtu.edu.cn

High-grade serous carcinoma (HGSC) represents a formidable challenge in the realm of ovarian cancer, notorious for its elusive early detection, poor prognosis and limited understanding of the intricacies of its pathogenesis. Over the past decade, genomic [1] and proteomic [2] approaches have provided glimpses into the biology of ovarian cancer, offering important clues for understanding the molecular mechanisms of HGSC. However, research on metabolomics, especially the study of metabolic changes in HGSC progression, remains relatively underexplored. Given the clinical urgency, there is a pressing need for innovative approaches to unveil the mysterious metabolic dynamics that underlie the initiation and progression of HGSC.

The paper entitled “Space- and Time-Resolved Metabolomics of a High-Grade Serous Ovarian Cancer Mouse Model”, recently published in *Cancers*, embarks on an innovative journey by combining time-resolved serum metabolomics with spatial lipidome profiling [3]. This novel approach offers a multifaceted perspective, elucidating the interplay between temporal and spatial alterations during HGSC’s tumultuous journey.

To delve deeper into these insights, the researchers employed a triple-mutant (TKO) HGSC mouse model. This unconventional approach allowed them to examine the temporal evolution of serum metabolites and lipids from 8 weeks of age until death and/or ascites formation, spanning a 10-month period. This meticulous timeline unveiled the transformation of metabolites, providing dynamic insights into the intricate changes accompanying HGSC progression. The analysis covered various metabolite classes, including 17 lipid subclasses, amino acids and tricarboxylic acid (TCA) cycle intermediates. These temporal patterns not only represent a comprehensive roadmap of metabolic rewiring during HGSC advancement, but also hold potential as early stage biomarkers and therapeutic targets.

More specifically, the investigation into alterations in the lipidome of HGSC using ultrahigh performance liquid chromatography-mass spectrometry (UHPLC-MS) led to the identification of a comprehensive range of metabolite species, including fatty acids, glycerophospholipids, lysoglycerophospholipids, carnitines, glycerolipids, sphingolipids, sterol lipids and polar metabolites such as arginine, citrulline and ribose-5-phosphate. Additionally, a ratio-based approach was employed to analyze lipid metabolites, offering a biologically meaningful perspective and the potential for diagnostic biomarkers. Specific lipid ratios, such as ceramide Cer(d18:1/18:0) and phosphatidylcholine PC(O-38:4), show promise as ovarian cancer diagnostic markers [4]. Furthermore, the use of ultrahigh-resolution matrix-assisted laser desorption/ionization (MALDI) mass spectrometry for spatial lipidome profiling revealed intricate lipid distributions within the reproductive system of the TKO mouse model. This innovative approach bridges the gap between systemic and localized metabolic changes, enhancing our understanding of disease progression.

The study underscores the pivotal role of metabolic reprogramming in driving HGSC’s trajectory. Core metabolic pathways, including lipid and fatty acid metabolism, amino acid biosynthesis, tricarboxylic acid (TCA) cycle activity and ovarian steroidogenesis, undergo intricate remodeling as HGSC advances. These adaptations align with the well-established landscape of cancer metabolism, where rapid cell proliferation fuels an increase in nutrient demands and energy generation. These metabolic shifts converge with changes in energy metabolism, mitochondrial and peroxisomal function, redox balance and inflammatory responses, collectively nurturing a conducive microenvironment for tumor growth. Inflammation plays a crucial role in ovarian cancer development and progression [5]. Alterations in the linoleic acid and arachidonic acid pathways observed in the progression of mouse HGSC raise questions regarding whether these changes are causative factors or consequences of inflammation, thereby necessitating further in vitro investigations to elucidate the underlying molecular mechanisms.

While this study offers enlightening insights, it does face limitations in metabolite coverage due to the ionization techniques in spatial lipidomics experiments. Additionally, the focus on advanced tumor tissues prompts questions with regard to spatial lipid distribution and transformations within benign tumors during the early stages of HGSC. The authors acknowledge these limitations and call for future investigations that encompass diverse ovarian cancer stages and incorporate various imaging modalities to refine our comprehension.

The TKO mouse model holds immense significance due to its accurate representation of human HGSC as well as the controlled experimental conditions it offers. This model has proven to be a valuable tool for studying HGSC and has provided valuable insights into its pathogenesis and potential treatment approaches [6,7]. The significance of the TKO mouse model is rooted in its fidelity to human HGSC as well as its controlled experimental conditions. However, as the research transcends species boundaries, bridging the gap between TKO mice and their human counterparts remains pivotal. This cross-species validation is not merely a verification of research methodology, but also holds the potential to reshape the clinical landscape, offering insights that can revolutionize diagnostic and therapeutic strategies.

In the backdrop of the relentless impact of ovarian cancer on women’s health, the paper “Space- and Time-Resolved Metabolomics of a High-Grade Serous Ovarian Cancer Mouse Model” unfurls a captivating tapestry of metabolic dynamics. The fusion of time-resolved serum metabolomics with spatial lipidome profiling yields insights that are as intricate as they are transformative. These revelations extend beyond the realms of laboratory walls, promising the discovery of hidden biomarkers and new therapeutic horizons. In this journey toward unraveling the intricate metabolic choreography orchestrated by HGSC, this research emerges as a beacon of hope, illuminating pathways that hold the potential to revolutionize the way we perceive and combat this enigmatic disease.
